# Tuning the Charge Transport in Nickel Salicylaldimine Polymers by the Ligand Structure

**DOI:** 10.3390/molecules27248798

**Published:** 2022-12-12

**Authors:** Daniil A. Lukyanov, Vladimir V. Sizov, Alexey I. Volkov, Evgenii V. Beletskii, Andrey N. Yankin, Elena V. Alekseeva, Oleg V. Levin

**Affiliations:** 1 Institute of Chemistry, Saint Petersburg University, 199034 St. Petersburg, Russia; 2 School of Physics and Engineering, ITMO University, Kronverksky Pr. 49A, 197101 St. Petersburg, Russia

**Keywords:** conductive polymers, conductivity, polaron, DFT, coordination, metalorganic polymers

## Abstract

The conductivity of the polymeric energy storage materials is the key factor limiting their performance. Conductivity of polymeric NiSalen materials, a prospective class of energy storage materials, was found to depend strongly on the length of the bridge between the nitrogen atoms of the ligand. Polymers obtained from the complexes containing C_3_ alkyl and hydroxyalkyl bridges showed an electrical conductivity one order of magnitude lower than those derived from more common complexes with C_2_ alkyl bridges. The observed difference was studied by means of cyclic voltammetry on interdigitated electrodes and operando spectroelectrochemistry, combined with density functional theory (DFT) calculations.

## 1. Introduction

Polymeric materials based on salen-type complexes with transition metals are widely used in energy storage devices [1,2,3,4,5,6,7,8,9,10], photoelectrochemical [11,12] and electrochromic systems [13,14], electrochemical sensors [15,16,17], and battery safety elements [18,19], due to their intrinsic conductivity [20] and redox properties [3,21]. Such materials could be easily deposited in a controllable manner directly on the electrode surface by electrochemical oxidative polymerization from the solution of a monomer [3]. The obtained films can be further reversibly oxidized and reduced, and high conductivity of such films ensures the fast rate of these processes [20], which is extremely important for most of the proposed applications.

**Figure 1:**
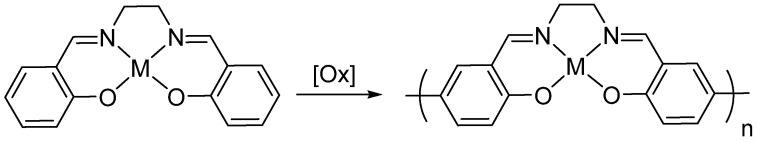
Schematic illustration for the oxidative polymerization of MSalens.

Oxidative electropolymerization of bis(salicylideniminato) complexes with transition metals (MSalens) is found, with rare exceptions [22], to proceed via the formation of C-C bonds between the 5-positions of phenyl rings [23,24], resulting in the formation of 4,4′-dihydroxybiphenyl fragments, held together by an imine bridge and metal center (Figure 1). The latter may provide conjugation via its d-orbital, providing the conductivity pathway along the polymer chain, typical for classic conductive polymers. MSalen-type polymers are generally considered as narrow band gap p-type semiconductors, for which electronic properties can be tuned in a wide range by the modification of the ligand structure and the choice of the metal center [25]. The charge transport in the MSalen-type polymer films is a complex process, involving different types of the delocalized and localized charge carriers and ionic transport, which makes it impossible to isolate the electronic conductivity from the charge diffusion determined by electrochemical methods such as electrochemical impedance spectroscopy. The effect of the ligand structure on the electrical conductivity of the MSalen-type polymers is also complex and poorly studied [26,27].

**Figure 2:**
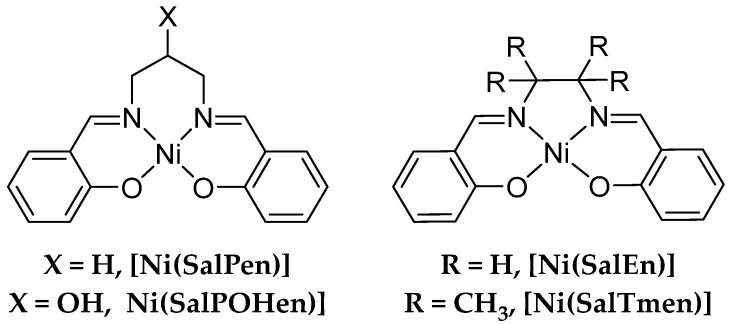
Structure of NiSalen complexes employed in this work.

In this work, we provide further insight on the conductivity of the NiSalen-type polymer films. For this purpose, a series of NiSalen-type complexes bearing different structural features and the conducting polymers derived thereof (Figure 2) was investigated. The variety served to examine the influence of the structural factors on the electrochemical behavior and electrical conductivity. The series consists of “C_2_-bridged” complexes [Ni(SalEn)] and [Ni(SalTmen)], as well as “C_3_-bridged” complexes [Ni(SalPen)] and [Ni(SalPOHen)].

## 2. Results and Discussion

### 2.1. Electrochemical Studies

**Figure 3:**
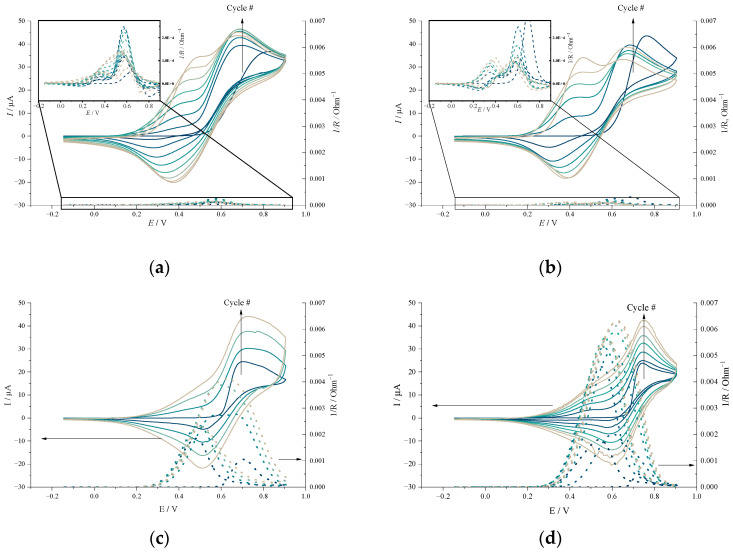
Cyclic voltammograms of electropolymerization and conductance of the films measured during the electrodeposition process of (**a**) [Ni(SalPOHen)], (**b**) [Ni(SalPen)], (**c**) [Ni(SalEn)] and (**d**) [Ni(SalTmen)], 1 mM monomer and 0.1 M LiClO_4_ solution in CH_3_CN, scan rate 5 mV s^−1^.

Electropolymerization of NiSalen-type polymers occurs when a monomer molecule is subjected to a potential positive enough to oxidize it, forming a radical cation. Such oxidation was observed as an irreversible peak on the cyclic voltammogram of monomer solution (Figure 3). In the first deposition cycle, the monomer oxidation peak was found at 0.831 V for [Ni(SalPOHen)], 0.761 V for [Ni(SalPen)], and 0.743 V for [Ni(SalEn)] and [Ni(SalTmen)]. In the second and following cycles, another pair of reversible peaks appeared, corresponding to the formation of electroactive polymer film. This process has little influence on the oxidation of the [Ni(SalEn)] and [Ni(SalTmen)] monomers, while the oxidation peaks of [Ni(SalPOHen)] and [Ni(SalPen)] shifted in the cathodic direction by more than 130 mV, becoming almost equal in potentials (0.695 V and 0.687 V, correspondingly). Such shift indicates that oxidation of complexes with three carbons in the diamine bridge is more facile on the surface of the formed polymer than their oxidation on an inert surface. It may result from strong interaction between monomers and the polymer. The growth rate of the polymer film can be estimated by the increase of the current of peak pair at ca. 500 mV, corresponding to the reversible redox processes in the formed film. Despite the monomer oxidation currents being close for all complexes, the growth of the [Ni(SalEn)]-based polymer is faster, and the same polymer reduction peak currents were obtained after four cycles for *poly*[Ni(SalEn)], six cycles for *poly*[Ni(SalPen)], seven cycles for *poly*[Ni(SalTmen)], and eight cycles for *poly*[Ni(SalPOHen)].

Operando analysis of the film conductance was carried out using the interdigitated electrodes array (IDE) technique (see Materials and Methods section for details). Monitoring of the conductivity on the IDE electrodes during electrodeposition revealed another important difference between the studied monomers (Figure 3). During the first cycle of [Ni(SalEn)] and [Ni(SalTmen)] polymerization, the conductance value remained close to zero till the beginning of the monomer oxidation peak. It confirms the absence of the polymer film or any other conductive pathways on the electrode surface. As soon as monomer oxidation starts (~0.6 V), the conductance increased, indicating the formation of a conductive polymer film. The conductance value remained more than 1 mS on the backward CV scan until the complete reduction of the polymer (~0.25 V). Then it dropped to an almost zero value, indicating that, along with many other conductive polymers, *poly*[Ni(SalEn)] and *poly*[Ni(SalTmen)] are insulators in the undoped (reduced) state. In the second cycle the conductance increased immediately with oxidation (i.e., doping) of the formed film. Upon the deposition of the next layer of the polymer, the conductance of the film increased, corresponding to the increase of the polymer thickness. The maximal values of conductance observed during polymerization were 4 mS for *poly*[Ni(SalEn)] and 6 mS for *poly*[Ni(SalTmen)]. During the electrodeposition of *poly*[Ni(SalPOHen)] and *poly*[Ni(SalPen)] films, the measured conductance did not exceed 0.2 mS in the whole polymer electroactivity range (0.25–0.5 V), which is one order of magnitude lower than the conductance of *poly*[Ni(SalEn)] and *poly*[Ni(SalTmen)].

The polymer films formed on the electrode can be reversibly oxidized and reduced in the monomer-free solution. The voltammograms of each film (Figure 3) show one pair of wide peaks. Similar to monomers in deposition solutions, C_3_-bridged polymers were oxidized at lower potentials than C_2_-bridged ones. Both C_3_-bridged polymers showed almost identical voltammetric responses with peak potential at *E*_c_ = 0.425 V, *E*_a_ = 0.571 V, *E*_1/2_ = 0.498 V for *poly*[Ni(SalPOHen)], and *E*_c_ = 0.439 V, *E*_a_ = 0.531 V, *E*_1/2_ = 0.485 V for *poly*[Ni(SalPen)], correspondingly. The voltammograms of the C_2_-bridged polymers contain a pair of main peaks located at more positive potentials, *E*_a_ = 0.695 V, *E*_c_ = 0.543 V, *E*_1/2_ = 0.619 V for *poly*[Ni(SalEn)] and *E*_a_ = 0.713 V, *E*_c_ = 0.603 V, *E*_1/2_ = 0.658 V for *poly*[Ni(SalTmen)], and shoulders at potentials of ca. 0.5 V.

**Figure 4:**
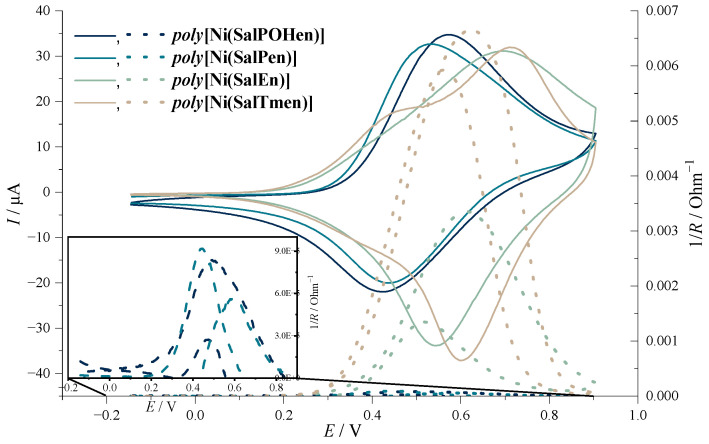
Cyclic voltammograms and operando conductance of poly[Ni(SalPOHen)], poly[Ni(SalPen)], poly[Ni(SalEn)] and poly[Ni(SalTmen)], 0.1 M LiClO_4_ in CH_3_CN, scan rate 5 mV s^−1^.

More surprising difference between the C_2_- and C_3_-bridged polymers can be found if we measure the conductance of the polymer films (Figure 4). All *poly-*NiSalen films were not conductive in the reduced form of the polymer (at potentials below 0.2 V). However, as soon as the polymer oxidation starts, the conductance of the *poly*[Ni(SalEn)] and *poly*[Ni(SalTmen)] films rapidly increased. It reached its maximum value at 0.610 V for *poly*[Ni(SalEn)] and 0.626 V for *poly*[Ni(SalTmen)], which is very close to the anodic peak potential of the polymer. During the subsequent oxidation of the polymer, the conductance of the film decreased, becoming negligibly small when the film was fully oxidized (~1 V). The following reduction of the film led to an increase of its conductance, which reached its maximal value at 0.474 V. This value, however, was more than five times less than the maximal polymer conductance, observed at the anodic branch of the voltammogram. The difference in conductance for the positive and negative sweeps was reproducible in subsequent voltammetric scans and does not, therefore, result from the degradation of the polymer. Such dependence of conductance on potential is typical for conductive polymer films [28]. Higher maximum conductance for the positive sweep than for the negative sweep is explained by Coulomb repulsion, which comes into effect when the polymer is highly oxidized. This repulsion can drive the polymer chains to adopt conformations in which the more localized charges are stabilized by separate segments, resulting in these segments being unengaged in the charge transport when its oxidized form is being reduced [29].

The shape of conductance-potential curves for *poly*[Ni(SalPOHen)] and *poly*[Ni(SalPen)] films resembled the curve observed for *poly*[Ni(SalEn)], however, their conductance values were more than one order of magnitude lower and approach values typical for the redox polymers on the same IDEs (Figure 4). On the contrary, conductance of *poly*[Ni(SalTmen)] was about two times higher than for *poly*[Ni(SalEn)], which indicates that the steric hindrance of NiSalen units, caused by CH_3_– groups of the *poly*[Ni(SalTmen)] bridge fragment, does not significantly affect the conductance of the derived polymeric material. Consequently, the abnormally low conductance of the *poly*[Ni(SalPOHen)] and *poly*[Ni(SalPen)] films was clearly caused by a C_3_ bridge of both complexes.

The degree of electronic coupling between the biphenyl fragments of the ligand and thus delocalization of charge carriers along the chain of the conductive polymer was estimated with the aid of cyclic voltammetry. The degree of delocalization in the NiSalen-type complexes is known to correlate with the difference between two oxidation peaks in CV, Δ*E*_ox_ [30,31]. Higher values of Δ*E*_ox_ indicate the delocalization of single oxidized states with the formation of polarons [27]. Indeed, the CV of *poly*[Ni(SalTmen)] showed significant Δ*E*_ox_, which indicates high delocalization along the polymeric chain. In the CV of *poly*[Ni(SalEn)], the oxidation peaks overlapped, resulting in a single broadened and unsymmetrical peak. Finally, the CVs of [Ni(SalPen)] and [Ni(SalPOHen)] showed only one anodic peak so the value Δ*E*_ox_ was negligible.

### 2.2. Crystal Structure

**Figure 5:**
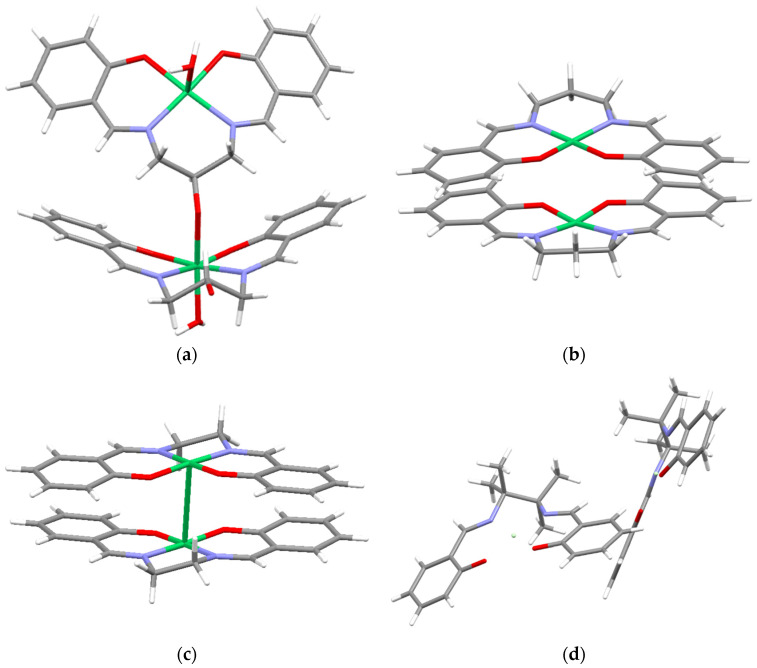
Crystal structures of dimeric units of (**a**) [Ni(SalPOHen)], (**b**) [Ni(SalPen)] [32], (**c**) [Ni(SalEn)] [33], and (**d**) [Ni(SalTmen)] [34].

To estimate the packing of the *poly-*NiSalen films, the X-ray crystal structures of the parent monomers were examined. The molecules of the [Ni(SalEn)] complex in crystal are stacked in the columnar structure (Figure 5a), as well as those of the homologous complex [Ni(SalPen)]. As anticipated, the bulky [Ni(SalTmen)] complex is packed in crystal without any stacking motif. The crystal structure of [Ni(SalPOHen)] possesses a unique feature: the molecules are arranged in a coordination polymer by binding of a bridge-appended OH group to the axial position of Ni center of another molecule, preventing stacking.

One may expect the packing of rigid oligomeric chains of *poly-*NiSalen to correlate with crystals of the corresponding monomers, which do not exhibit any dependence of the bridge length itself and rather tend to be affected by the steric effects and intermolecular coordination.

### 2.3. UV-Vis Spectroscopy

**Figure 6:**
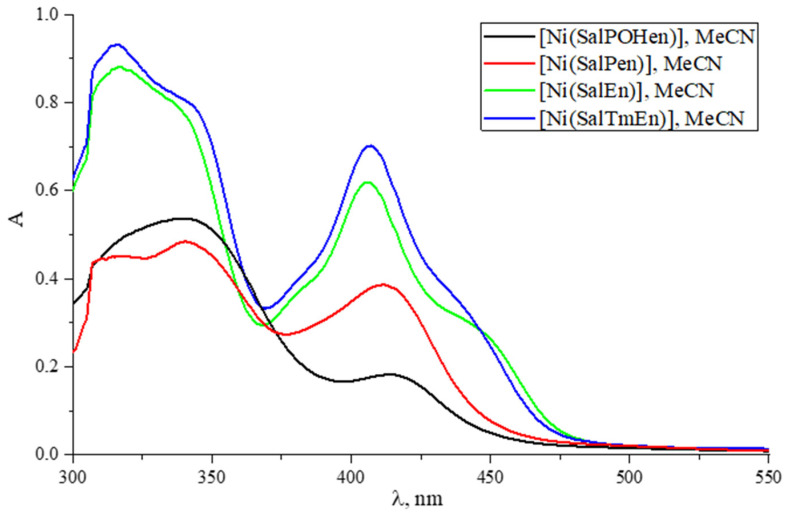
UV-Vis spectra of [Ni(SalPOHen)] (black), [Ni(SalPen)] (red), [Ni(SalEn)] (green) and [Ni(SalTmen)] (blue), 0.1 mM in CH_3_CN.

Ultraviolet–visible (UV-Vis) spectra of the complexes [Ni(SalPen)] and [Ni(SalPOHen)] in CH_3_CN differed from those of the reference complexes [Ni(SalEn)] and [Ni(SalTmen)] (Figure 6). The latter two showed neatly the same spectra with maxima at ca. 320 nm and 410 nm with shoulders near 340 nm and 435 nm, respectively. The spectrum of [Ni(SalPen)] had the same signals around 320 nm, 340 nm, while the low energy signal suffers ca. 10 nm red shift and a drop in absorbance, with no shoulder at ca. 435 nm. The high energy absorbance of [Ni(SalPOHen)] was nearly the same as the non-hydroxylated analog, but the low energy band was much less pronounced and was shifted to 430 nm. These facts are consistent with the notion that the bands at 320–340 nm were attributed to the intraligand transitions (ILCT), and so remain at the same positions with different bridges, while the absorption bands around 410–430 nm were traditionally interpreted as metal-to-ligand charge transfer (MLCT) transitions, which suffer from the factors affecting the Ni center [35]. Consequently, the observed spectral differences between C_2_- and C_3_-bridged monomers are caused by the different coordination states of Ni.

The operando UV-Vis spectroelectrochemical technique is a common tool to study the stages of oxidation and reduction of conductive polymer films. Spectroscopic features of NiSalen-type polymers in different electronic and oxidative states are well described [30,36,37], providing a lot of data for comparison. As shown in our previous work, the electrochemical properties of polymeric complexes of nickel with salen-type ligands are governed by the orbitals of both ligand and central metal atom [38].

**Figure 7:**
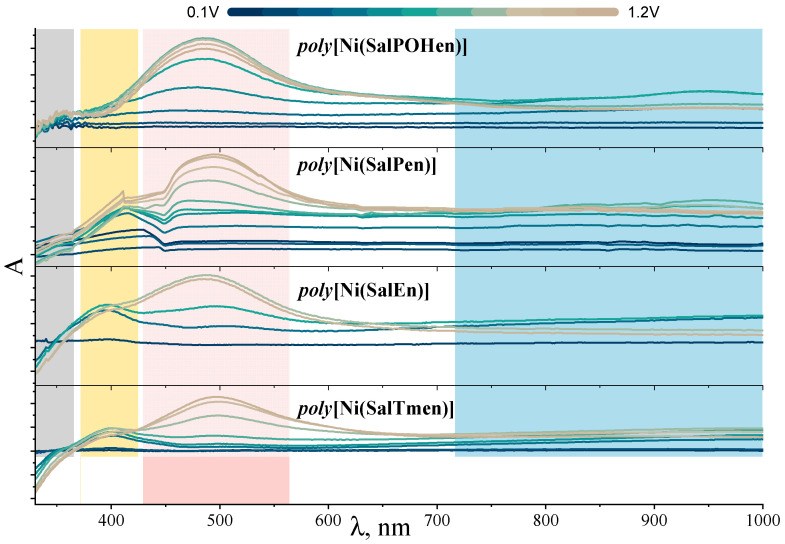
Operando UV-vis spectra of poly[Ni(SalpOHen)], poly[Ni(SalPen)], poly[Ni(SalEn)] and poly[Ni(SalTmen)] films at various potentials with the reduced form (at 0.0 V) subtracted.

A series of operando UV-Vis spectra for the films of four polymeric complexes at different applied potentials were recorded. The subtraction of the spectrum of the reduced form of the film (i.e., the spectrum obtained for the film at 0 V) from the spectra of the film at various oxidation potentials allows to determine the growth of bands intensities depending on the degree of oxidation of the film (Figure 7).

The behavior of the poly[Ni(SalEn)] and poly[Ni(SalTmen)] films during oxidation is consistent with literature data [36]. The bands at 320 nm, 400 nm, 485 nm, and the broad near-infrared (NIR) band below 1000 nm changed during the first stage of the oxidation. The bands at 400 nm and >1000 nm increased along with the increase of the potential, while the band at 320 nm decreased simultaneously. At the second stage of the oxidation, a rapid growth of the band at ca. 490 nm occurred. In contrast, *poly*[Ni(SalPen)] and, especially *poly*[Ni(SalPOHen)], showed subtle changes at 320 nm and 400 nm upon oxidation. Instead of this, a new broad band at ca. 900 nm emerged during the first stage of oxidation, accompanied by the immediate growth of the band at 490 nm.

**Figure 8:**
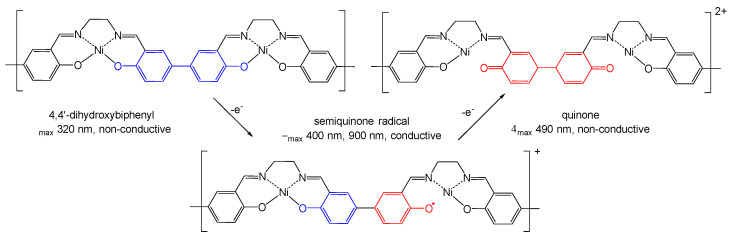
Schematic illustration of ligand oxidation in poly-NiSalens.

Growth of the low-energy band with λ > 1000 nm during the oxidation indicate the formation of polarons, which are responsible for the conductance along the polymer chain [36]. On a molecular level (Figure 8), the polarons correspond to a single oxidized NiSalen unit (radical cation). Upon the further oxidation, polarons are substituted by bipolarons, which correspond to a double oxidized NiSalen unit (dication) [26]. Oxidation of NiSalen complexes is known to process as ligand oxidation [30], which matches with the spectroelectrochemical results. The absorbance at 320 nm, which is ceased upon oxidation, corresponds to the 4,4′-dihydroxybiphenyl fragment of the ligand. The absorbance at 400 nm, appearing at moderate degrees of oxidation, is characteristic of localized semiquinone radicals of 4,4-dihydroxybiphenyl [39]. The formation of quinone fragments upon further oxidation leads to the appearance of the band at 490 nm [40].

The low intensity of absorption at 400 nm and early appearance of the band at 490 nm in C_3_-bridged complexes are due to the low comproportionation constant for the semiquinone fragments of these complexes, resulting in the formation of the quinone fragments rather than semiquinone radicals. In addition, the NIR band responsible for the polaronic absorption shifts from >1000 nm for C_2_-bridged complexes to ca. 950 nm for the C_3_-bridged ones, indicating the poor delocalization of the latter, which is likely caused by the distortion of the square planar geometry of the metal center and less efficient overlapping of the Ni d_xz_ orbital and π-orbitals of the ligands [37].

### 2.4. DFT Calculations

**Figure 9:**
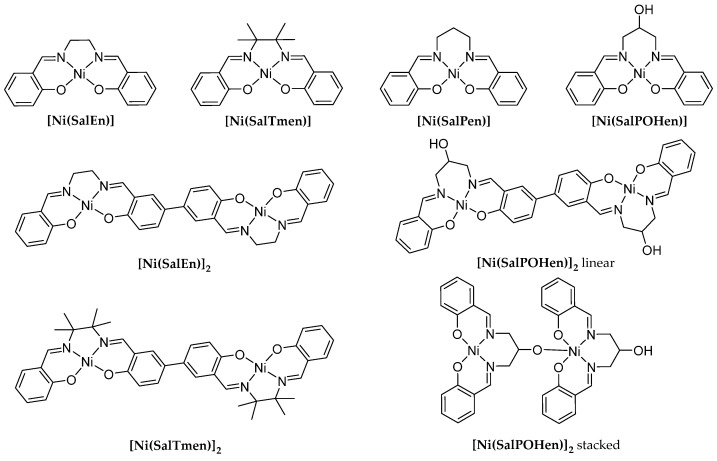
Objects of the computational study.

A quantum chemical density functional theory (DFT) study was carried out to gain molecular-level insight into the structural and electronic properties of the complexes under consideration. This study pursued two primary goals. First, calculations were performed for monomer units and stack-like dimers of all four complexes to provide the description of their UV-Vis spectra and to estimate possible systematic errors by comparing the computational data with experimental measurements. Second, calculations were carried out for dimers, in which the monomer units were connected by a C–C bond. These dimers served as simplified models of coordination polymers formed upon electropolymerization (Figure 9).

Geometry optimizations for [Ni(SalEn)] and [Ni(SalTmen)] did not yield any unexpected results: both complexes were shown to be low-spin species with structural parameters close to those reported in the literature by various experimental and computational studies. The calculated energy for low-spin structures of [Ni(SalEn)] and [Ni(SalTmen)] was lower than that for the high-spin species by 44 kJ mol^−1^ and 46 kJ mol^−1^, respectively. In contrast to that, optimization of the neutral [Ni(SalPOHen)] complex suggested that the high-spin system was marginally more stable (by ca. 9 kJ mol^−1^) than the low-spin one. For [Ni(SalPen)], the low-spin structure had lower energy, though the difference between low-spin and high-spin species (18 kJ mol^−1^) was significantly less than that for [Ni(SalEn)] and [Ni(SalTmen)]. As a result, the high-spin form of the [Ni(SalPOHen)] complex was likely to be more abundant, while high-spin [Ni(SalPen)] can also be present in solution, though in significantly smaller amounts. In reality, both low- and high-spin species are likely to be present under reasonable conditions, and the exact composition of this mixture is likely to be heavily influenced by environment-related factors, such as the nature of the solvent, the presence of background electrolytes, etc. In the solid state, as shown by X-ray structural data, [Ni(SalPOHen)] appeared to exist in the low-spin state, however, still bent significantly in comparison with other complexes, both C_2_- and C_3_-bridged. This can be attributed to somewhat better packing, which can be achieved for the low-spin structure, and the energy thereby gained may be significantly larger than the energy difference between the two forms of [Ni(SalPOHen)]. However, the observed paramagnetic effects in the ^1^H NMR spectra of [Ni(SalPOHen)] in DMSO-*d_6_* (Figure S1) indicate the presence of high-spin species in the solution.

**Figure 10:**
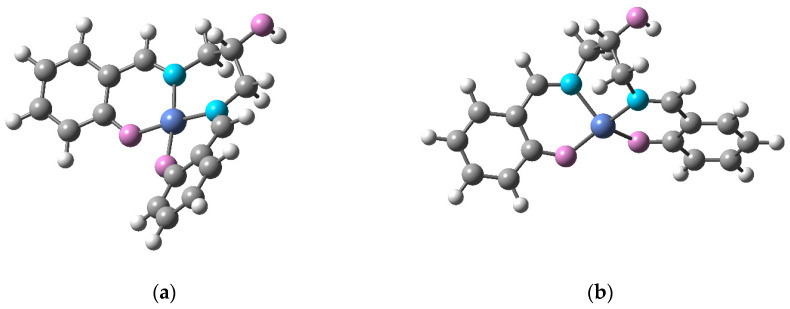
Geometries of (**a**) low-spin and (**b**) high-spin forms of [Ni(SalPOHen)].

The structures for high-spin and low-spin species were significantly different. While the structures of the low-spin complexes were significantly non-planar (the angle between the planes of two phenyl rings was ca. 32 degrees), they displayed the same square-planar coordination mode for the metal center, which is generally typical for low-spin Ni(II) complexes, including [Ni(SalEn)] and [Ni(SalTmen)]. However, the structure of the high-spin complex was bent unsymmetrically, and the coordination environment of the nickel atom could be described as a distorted tetrahedron (Figure 10). For C_3_-bridged complexes, this structural transformation is facilitated by a longer and more flexible bridging fragment, as compared to the C_2_-bridged complexes.

The structures of stack-like dimers were obtained from DFT geometry optimizations. However, only for [Ni(SalEn)]_2_ did the stack-like dimer have a plane-parallel structure, which may suggest the presence of stacking interactions. A Ni–Ni distance of 3.39 Å was observed for this complex, which is very close to the experimental value of 3.33 Å obtained from X-ray single-crystal measurements. For [Ni(SalTmen)]_2_, the calculations reproduce the general layout of the dimer and the Ni–Ni distance (calculated distance of 6.40 Å vs. 6.47 Å in X-ray experiment), though the mutual orientation for an isolated pair of molecules was somewhat different from the tightly packed crystal. Considering that [Ni(SalTmen)] oligomers, despite the lack of the interchain stacking, show very high conductivity, allows us to reveal the minor role of the intrastack charge transport in the whole material conductivity. Finally, for [Ni(SalPOHen)]_2_, the calculations failed to reproduce the structural motif characteristic of this complex in the solid state: in the optimized dimer the molecules were buckled in the same direction rather than in opposite directions, as the experimental data suggest. This was caused by the inherent limitations of the model, which considered only two molecules and disregarded interactions with other molecules. On the other hand, in solutions the structures formed because of possible spontaneous aggregation were likely to be closer to those obtained in calculations than to those observed experimentally for the solid state.

The most interesting feature of [Ni(SalPOHen)] molecules is their ability to form hydrogen bonds with each other and with other molecules containing suitable fragments, such as OH-groups. As compared to non-bonded dimer formation for [Ni(SalEn)], for [Ni(SalPOHen)], the energy of formation, which was estimated as the difference between the energy of the dimer and the sum of energies of two monomers, was somewhat lower: −72 kJ mol^−1^ for [Ni(SalEn)] vs. −43 kJ mol^−1^ for [Ni(SalPOHen)]. However, for the former complex the stack-like [Ni(SalEn)]_2_ dimer has almost the maximum possible area of contact, which results in a very large contribution of attractive dispersion forces. In contrast to that for [Ni(SalPOHen)]_2_, the area of contact of two molecules in the dimer was small, but the formation energy was only 40% lower than for [Ni(SalEn)], which can be attributed to a significant energy benefit due to the formation of a hydrogen bond between OH-groups of the monomers.

The presence of two unpaired electrons at the metal center favors the attachment of axial ligands to NiSalen complexes, so the high-spin [Ni(SalPOHen)] presents the most likely target for solvent coordination. Furthermore, the structure of the bridging fragment also makes [Ni(SalPOHen)] more susceptible to coordination of solvent molecules than [Ni(SalEn)] or [Ni(SalTmen)]. Of particular interest, is the behavior of [Ni(SalPOHen)] in isopropyl alcohol, as the molecular structure of this solvent closely resembles that of the bridging fragment in the SalPOHen ligand. As a result, one of the possible structures formed upon coordination of *i*-PrOH resembles that of stack-like [Ni(SalPOHen)]_2_ dimer with the OH-group of the alcohol molecule achieving a close contact with the nickel atom. However, at least two more possible structures were identified by geometry optimizations, in which the OH-group of the solvent was attached to the same group in the bridging fragment of the complex. The formation energies of all three [Ni(SalPOHen)]*i-PrOH structures were within the margins of computational error, so all these structures may be present in solution in comparable concentrations along with stack-like [Ni(SalPOHen)]_2_ dimers.

The structural parameters obtained from DFT geometry optimizations for chain-like (NiSalen)_2_ dimers were generally in line with the observations previously reported for oligomers of [Ni(SalEn)] [38]. In the neutral dimers, the monomeric units were rotated relative to each other, with the dihedral angle between the planes of two phenyl rings adjacent to the C–C bond, which is formed between monomers in the dimer, being 40–41 degrees for all three complexes. For the oxidized chain-like (NiSalen)_2_^2+^ dimers the angle between the rings decreased to only 4–5 degrees for [Ni(SalEn)]_2_^2+^ and [Ni(SalTmen)]_2_^2+^, while for [Ni(SalPen)] and [Ni(SalPOHen)]_2_^2+^ the angle was very close to zero. These structural changes were accompanied by the shortening of C–C bonds between the monomeric units from 1.48 Å for neutral [Ni(SalEn)]_2_ complexes to 1.39 Å for [Ni(SalEn)]_2_^2+^ dimers. This indicates an effective conjugation between the phenolate fragments of the neighbor monomeric unit upon oxidation.

The UV-visible spectra obtained from the TD-DFT calculations for all low-spin monomer complexes are very similar (Table 1), featuring a low-energy transition at 346–347 nm followed by higher-energy transitions at 275–276 nm and a variety of photophysically active excited states with wavelengths lower than 250 nm, which form a single structured band due to small intervals between individual transitions. According to natural transition orbitals (NTO) analysis, all these bands could be described as ILCT with a certain fraction of MLCT. The only notable exclusion was the transition at 230 nm for [Ni(SalPOHen)], which has a significant contribution of ligand-to-metal charge transfer (LMCT) rather than MLCT. A comparison with the experimental data suggested that the transition energies were systematically overestimated by up to 0.5 eV, though the analysis of possible changes between the complexes was still quite feasible.

An important computational result was the difference between the spectra of high-spin and low-spin forms of [Ni(SalPen)] and [Ni(SalPOHen)] complexes. For the high-spin species, the spectrum lacked the transitions at 346 nm and 275 nm, which in practice would result in the total absence of the distinct low-energy absorption band. This feature might explain the experimentally observed behavior of [Ni(SalPOHen)] in acetonitrile, though the low-energy absorption does not disappear completely due to the simultaneous presence of high-spin and low-spin forms in the solution.

However, the existence of high-spin species may be only one of the factors responsible for the diminishing of low-energy absorption for [Ni(SalPOHen)]. Another important point is the difference in spectral properties of the monomer [Ni(SalPOHen)] and stack-like [Ni(SalPOHen)]_2_. Even in the low-spin state, the latter complexes might affect the adsorption intensity in the lower-energy region: though for the dimer the oscillator strength for transition at 346 nm increases to 0.28, it is likely to result in a noticeable decrease of the observed absorbance if the extinction is measured per mole of monomer units.

UV-visible spectra were obtained from TD-DFT calculations for chain-like dimers of all four complexes to provide the assignment for the observed spectral changes upon the oxidation of complexes. The neutral dimers are subject to the same high-spin vs. low-spin controversy, which has been observed for Ni(II) monomers. For oxidized (NiSalen)_2_^2+^ species this problem should not occur, as Ni(III) has only one unpaired electron. However, the presence of Ni(III) in oxidized complexes cannot be guaranteed, so the computational study of (NiSalen)_2_^2+^ is even more challenging and time-consuming than for (NiSalen)_2_^0^ due to the substantially greater number of possible electronic structure variants. The discussion below will address only dimers with the same pattern of electron and spin density distribution, though the favorability of such patterns may vary for individual complexes.

As compared to neutral chain-like dimers (Table 2), the one-electron-per-monomer oxidation (i.e., a total of two electrons are withdrawn from the dimer) leads to the emergence of low-energy bands at approximately 1350 nm, 660 nm, 475 nm, and 445 nm (Table 3). The first two of these bands are assigned to ligand-to-ligand charge transfer (LLCT) transitions from the outer pair of phenyl rings to the two rings adjacent to the C–C bond between the monomers. Absorption at 475–480 nm, which can also be described as LLCT, has a somewhat different nature: the NTO for this transition is more or less uniformly distributed over the entire length of the dimer, while the NTO* orbital is predominantly localized on the inner pair of phenyl rings. The interpretation of the transition at 444–446 nm is different for [Ni(SalEn)]_2_^2+^ and [Ni(SalTmen)]_2_^2+^. For the latter complex, the nature of this transition is very similar to that for the one observed at 475 nm, while for the former species, the transition is almost completely localized at the inner pair of rings and does not involve the peripheral regions of the dimer. This interpretation of electronic spectra for oxidized dimers is generally in line with the one reported earlier [38]. One should keep in mind that, as in the TD-DFT calculations for monomer complexes, the positions of absorption bands were significantly blue-shifted; for dimers (both neutral and charged) the same direction and magnitude of such shift can be expected when comparing the computational data to the experiment.

One of the most notable results of the present TD-DFT calculations was the absence of the 660 nm band for [Ni(SalPen)]_2_^2+^, and [Ni(SalPOHen)]_2_^2+^ (Table 2). This feature could have been assigned to the differences in the electronic structure of C_2_- and C_3_-bridged complexes. However, according to the NTO analysis, the nature of the corresponding transition does not appear to be directly connected to the nature of the bridging fragment, as this transition is an LLCT one and is very similar to the lower-energy transition at ca. 1350 nm, which was observed in computed electronic spectra of all four complexes. A more detailed comparison of the excited states obtained from TD-DFT calculations, including inactive excited states, is provided in Table 4 for [Ni(SalTmen)]_2_^2+^ and [Ni(SalPOHen)]_2_^2+^. One can see that the two lowest singlets (1355/1371 nm and 1235/1278 nm) were very similar for these two complexes, but the order of higher-energy states was significantly different. For [Ni(SalPOHen)]_2_^2+^, it was problematic to identify unambiguously a direct counterpart of the active singlet state observed for Ni(SalTmen)]_2_^2+^ at 654 nm; however, for the latter complex this state was the third lowest singlet (S_3_), while for the former compound it could be the fifth (S_5_) or even higher (counting the degenerate singlet at 687 nm as two states).

The electronic structure of oxidized (NiSalen)_2_^2+^ species, which were considered as simplified models of polymeric complexes occurring in poly(NiSalen) films, is likely to be responsible for the observed difference in conductivity between C_2_- and C_3_-bridged polymers. However, as electric conductivity is a dynamic property, there are no obvious descriptors yielding information on conductivity from stationary DFT calculations.

Though the conductivity of complexes under investigation cannot be evaluated by straightforward DFT calculations, one can use the computed energy difference between the highest occupied (HOMO) and lowest unoccupied (LUMO) orbitals as a crude estimate of the conductive ability of the molecule. For neutral (NiSalen)_2_ complexes, the HOMO/LUMO gap was about 5.5 eV, which is only marginally lower than for NiSalen monomers (ca. 6 eV). One-electron oxidation of the dimers (i.e., withdrawal of a half-electron per monomeric unit) does not significantly improve the conducting ability, as the HOMO/LUMO gap decreased to only 2.9–3.0 eV. Finally, for (NiSalen)_2_^2+^ species, the calculated HOMO/LUMO gap diminished to ca. 2 eV, which can be regarded as a potential indicator of efficient electronic conductivity. In this respect the complexes with trimethylenediamine-based bridging fragments ([Ni(SalPen)] and [Ni(SalPOHen)]) are quite similar to complexes with ethylenediamine-based bridges ([Ni(SalEn)] and [Ni(SalTmen)]), because the HOMO/LUMO energy difference for [Ni(SalPen)]_2_^2+^ and [Ni(SalPOHen)]_2_^2+^ is only slightly higher than for [Ni(SalEn)]_2_^2+^ and [Ni(SalTmen)]_2_^2+^ (2.04–2.05 eV vs. 1.96–1.98 eV). Therefore, from the computational point of view, C_2_-bridged polymeric complexes are likely to be more conductive, but the difference in HOMO/LUMO energies is too subtle to be the only factor determining the experimentally observed conductive properties.

## 3. Conclusions

We have shown that the bridge geometry of the NiSalen monomer determines the properties of the polymer obtained thereof. According to the results of operando conductance and spectroelectrochemical studies combined with DFT computations, we have found that the main factor that determines the charge transport along the polymer chain is the geometry and, thus, spin state of the Ni center of the monomeric unit. The C_2_ bridge is an optimal choice for the planar complexes with an effective π-conjugation, resulting in high electrical conductivity. In contrast, the C_3_ bridge favors non-planar and high-spin complexes, which, being incorporated into the polymeric chains, disrupt the charge transport pathways. In the C_2_-bridged NiSalen polymers, the polaronic charge transport deals the main contribution to the conductivity of the material, while in its C_3_-bridged analogs the polaronic transport is turned off, and the bulk conductivity is limited by the redox-hopping charge transport.

## 4. Materials and Methods

The NMR spectra were acquired on a Bruker Avance 400 MHz spectrometer. The residual solvent signal was used for calibration. The FTIR spectra were recorded on a Shimadzu IRAffinity-1 spectrometer in KBr pellets. The HRMS spectra were recorded on a Brucker maXis ESI-TOF spectrometer. The X-ray single crystal analysis was performed on an Agilent Technologies «Supernova» diffractometer with monochromated Mo Kα radiation at 100 K. The UV-Vis spectra were recorded on a Shimadzu UV-1700 dual-beam spectrophotometer in 1 cm quartz cuvettes.

Reagents and solvents of “reagent grade” from local suppliers and 1,3-diaminopropan-2-ol from abcr, Germany were used for the syntheses. Acetonitrile HPLC grade (CryoChrome, St Petersburg, Russia), anhydrous LiClO_4_ (Sigma-Aldrich, St. Louis, MO, USA) and Et_4_NBF_4_ (abcr, Karlsruhe, Germany) were used for electrochemical measurements as received. [Ni(SalEn)], [Ni(SalTmen)], and [Ni(SalPen)] were obtained according to the literature [24,41].

**1,3-bis(salicylidene)imino-2-hydroxypropane (SalPOHen)**. Salicylaldehyde (2.4 g, 2.1 mL, 10 mmol) and 1,3-diaminopropan-2-ol (0.45 g, 5 mmol) were dissolved in 10 mL of ethanol and stirred at reflux for 2.5 h and cooled down. The resulting yellow precipitate was filtered and dried in vacuo to obtain the SalPOHen (2.16 g, 7.2 mmol, 72%) as yellow crystalline solid.

Analytical data were consistent with those reported in literature [42].

**[Ni(SalPOHen)] complex**. To a SalPOHen (298 mg, 1 mmol) solution in 10 mL of hot EtOH aqueous ammonia (2 mL) was added, followed by a solution of Ni(OAc)_2_^*^4H_2_O (249 mg, 1 mmol) in 6 mL H_2_O. The mixture was stirred for 2.5 h, an emerald-colored precipitate was filtered and dried in vacuo at 140 °C, yielding the desired [Ni(SalPOHen)] (221 mg, 0.62 mmol, 62%) as a brown solid. The obtained product turns green on exposure to air and restores to a brown color upon drying at 140 °C.

IR (KBr) *ν*˜, cm^−1^: 1626 (C=N). ^1^H NMR (DMSO-d_6_, 400 MHz) *δ*, ppm: 16.8 (br.s, 2H), 11.70 (br.s, 2H), 8.97 (s, 2H), 7.39 (s, 2H), 6.57 (s, 2H), 6.44 (s, 2H), 5.48 (br.s, 1H), 3.98 (br.s, 1H). HRMS (ESI^+^/TOF) m/z: [M+H]^+^ Calcd for C_17_H_17_N_2_O_3_Ni^+^ 355.0587; Found 355.0571. Crystal of [Ni(SalPOHen)] suitable for X-ray analysis was grown by slow cooling of the solution in CH_3_CN immersed in hot water inside the Dewar flask. The file CCDC 1875699 contains the supplementary crystallographic data for this paper (see supplementary materials). These data can be obtained free of charge from The Cambridge Crystallographic Data Centre via http://www.ccdc.cam.ac.uk (accessed on 20 October 2022).

In electrochemical measurements, all potentials were presented against BASi-2062 Ag/AgNO_3_ electrode (~+0.4 V vs Ag/AgCl) unless stated otherwise. Bipotentiostat µSTAT 400 (DropSens, Oviedo, Spain) was used for electrochemical synthesis and studies. For cyclic voltammetry and conductance measurements, the film was synthesized and studied on an interdigitated electrode (IDE electrodes, DropSens IDEPT-5). The synthesis of *poly-*NiSalen films was performed by cyclic voltammetry method using two contacts of the interdigitated electrode as two working electrodes. Platinum wire was used as a counter electrode and Ag/AgNO_3_ electrode—as a reference one. The precursor solution contained 0.3 mg ml^−1^ of the monomer and 0.1 M LiClO_4_ in acetonitrile. The synthesis went on until the difference in currents of each consequent cycle was negligible, thus ensuring that the polymer had “grown” all the way between two electrodes and formed a continuous film.

Operando electrical conductance was measured on IDE electrodes (vide supra) using the difference CV technique, which was described in detail elsewhere [43]. Briefly, the IDE electrode was used which comprise of two interdigitated comb electrodes with 5 µm gap between the combs. Polymerization fills the gaps with the polymer, which thus act as a serial resistive element between the electrodes. The difference CV is then recorded using the bipotentiostat scheme with the two electrodes as working electrodes WE_1_ and WE_2_, with the constant difference in applied potentials (ΔE_1–2_ = 10 mV). Due to the low ΔE_1–2_, the Faradaic currents on both electrodes were assumed to be equal, and the Ohmic current between the WE_1_ and WE_2_ can be expressed as:

I_Ohm_ = I_1_ − I_2_, (1)

and thus, the conductance of the film can be calculated as

S = I_Ohm_/ΔE_1 − 2_. (2)

The operando UV-Vis spectra of the *poly-*NiSalen films were obtained while the film deposited on an indium-tin oxide (ITO) electrode was electrochemically oxidized in a 0.1 M Et_4_NBF_4_ solution in acetonitrile. A Pt wire was used as a counter electrode and Ag wire was used as a pseudo-reference electrode. The spectra were obtained in a 330 nm to 1100 nm range. Prior to registration of each spectrum, the system was brought into a stationary condition (i.e., constant current) under certain potential. First, the spectrum of the film at 0.0 V (vs Ag wire) was obtained. After that, spectra were obtained in +0.4 to +1.2 V potentials range with a +0.1 V step.

Quantum chemical computational study was carried out using density functional theory (DFT) calculations. The DFT geometry optimizations were carried out for NiSalen monomers and (NiSalen)_2_ dimers without imposing any restrictions on molecular symmetry or structural parameters; the resulting structures were verified by vibrational frequency analysis. The DFT calculations were performed using the CAM-B3LYP [44] long-range-corrected hybrid functional with Grimme’s D3 dispersion term [45] and 6-31+G* basis set for all atoms. The choice of the functional was largely determined by the necessity to perform calculations for stack-like dimers, for which the functional with a dispersion term and corrected long-range electrostatics was expected to provide a significantly more adequate description than other functionals. The relatively small basis set was justified by the axiomatic requirement to treat monomers and larger dimeric units within the framework of the same computational approach. In fact, preliminary test calculations have shown that the expansion of the basis set to 6-311+G*, which resulted in a ca. 20% increase in the number of basis functions, had only a minor effect on the optimized structures and computed UV-visible spectra of the complexes considered in this study. The UV-visible spectra were obtained from time-dependent DFT (TD-DFT) calculations; the nature of electronic transitions was analyzed using the natural transition orbitals (NTO) formalism [46].

All quantum chemical calculations were carried out using the Gaussian 16 package [47]. The NTO analysis of electronic transitions was completed with the help of the MultiWFN v. 3.5 software [48]. Visualization of molecular structures and surfaces was performed using GaussView 5.0 software [49].

## Figures and Tables

**Table 1:** Electronic spectra for [Ni(SalEn)], [Ni(SalTmen)], [Ni(SalPen)], and [Ni(SalPOHen)] obtained from TD-DFT calculations, λ (*f*). λ is the wavelength corresponding to an electronic transition, *f* is oscillator strength. Only transitions with oscillator strength of 0.1 or greater are shown.

[Ni(SalEn)]	[Ni(SalTmen)]	[Ni(SalPen)]		[Ni(SalPOHen)]	
Low-Spin	Low-Spin	Low-Spin	High-Spin	Low-Spin	High-Spin
347 (0.16)	347 (0.17)	345 (0.16)		346 (0.17)	
276 (0.14)	275 (0.12)				
250 (0.21)	249 (0.21)	248 (0.15)	250 (0.20)	249 (0.18)	248 (0.40)
238 (0.16)	239 (0.17)				
		233 (0.31)		233 (0.22)	
				230 (0.13)	
229 (0.38)	227 (0.54)	228 (0.53)	228 (0.13)	227 (0.43)	227 (0.23)

**Table 2:** Electronic spectra for [Ni(SalEn)]_2_^0^, [Ni(SalTmen)]_2_^0^, [Ni(SalPen)]_2_^0^, and [Ni(SalPOHen)]_2_^0^ obtained from TD-DFT calculations, λ (*f*). λ is the wavelength corresponding to an electronic transition, *f* is oscillator strength. Only transitions with oscillator strength of 0.1 or greater are shown.

[Ni(SalEn)]_2_^0^	[Ni(SalTmen)]_2_^0^	[Ni(SalPen)]_2_^0^		[Ni(SalPOHen)]_2_^0^	
Low-Spin	Low-Spin	Low-Spin	High-Spin	Low-Spin	High-Spin
379 (0.16)	374 (0.17)				
354 (0.22)	352 (0.22)	350 (0.21)	350 (0.11)	352 (0.22)	
286 (0.19)	284 (0.29)	292 (0.12)		291 (0.13)	
265 (0.82)	265 (0.80)	264 (0.76)	257 (0.36)	263 (0.74)	
259 (0.17)			256 (0.12)		
257 (0.47)	257 (0.77)	253 (0.15)	252 (0.43)	253 (0.19)	
254 (0.47)	253 (0.31)		250 (0.25)	250 (0.55)	
248 (1.04)	247 (0.71)	249 (1.95)	248 (0.29)	250 (1.45)	
241 (0.10)	246 (0.39)	248 (0.10)	244 (0.22)		
			241 (0.16)		
236 (0.42)	235 (0.57)	235 (0.15)	239 (0.31)	236 (0.16)	

**Table 3:** Electronic spectra for [Ni(SalEn)]_2_^2+^, [Ni(SalTmen)]_2_^2+^, [Ni(SalPen)]_2_^2+^, and [Ni(SalPOHen)]_2_^2+^ obtained from TD-DFT calculations, λ (*f*). λ is the wavelength corresponding to an electronic transition, *f* is oscillator strength. Only transitions with oscillator strength of 0.1 or greater are shown.

[Ni(SalEn)]_2_^2+^	[Ni(SalTmen)]_2_^2+^	[Ni(SalPen)]_2_^2+^	[Ni(SalPOHen)]_2_^2+^
1354 (0.61)	1355 (0.59)	1393 (0.39)	1371 (0.39)
661 (0.23)	654 (0.24)		
475 (0.84)	480 (0.78)	462 (1.26)	462 (1.25)
444 (0.20)	446 (0.28)	432 (0.12)	431 (0.12)
	351 (0.10)	356 (0.29)	356 (0.26)
342 (0.29)	344 (0.28)		
338 (0.13)	335 (0.13)	333 (0.13)	335 (0.13)
263 (0.20)			
260 (0.57)	260 (0.35)	256 (0.50)	258 (0.47)
245 (0.11)	256 (0.33)	249 (0.13)	253 (0.12)
	241 (0.18)	243 (0.17)	244 (0.30)
237 (0.17)	235 (0.15)	241 (0.13)	

**Table 4:** Low-energy singlet excited states for [Ni(SalTmen)]_2_^2+^ and [Ni(SalPOHen)]_2_^2+^, as obtained from TD-DFT calculations, λ (*f*). λ is the wavelength corresponding to an electronic transition, *f* is oscillator strength. * labels degenerate states, **bold** numbers highlight active transitions.

[Ni(SalTmen)]_2_^2+^	[Ni(SalPOHen)]_2_^2+^
**1355 (0.59)**	**1371 (0.39)**
1235 (0.00)	1278 (0.00)
	687 * (0.00)
**654 (0.24)**	675 (0.03)
	672 (0.00)
	634 (0.08)
639 (0.00)	630 (0.00)
557 * (0.00)	585 * (0.00)
538 * (0.00)	
527 * (0.00)	510 * (0.00)
492 * (0.00)	500 * (0.00)
**480 (0.78)**	**462 (1.25)**

## Data Availability

Not applicable.

## Supplementary Materials

The following supporting information can be downloaded at https://www.mdpi.com/article/10.3390/molecules27248798/s1, Figure S1: Operando UV-Vis spectra of the studied polymers; Figure S2: ^1^H NMR spectrum of [Ni(SalPOHen)]; Figure S3: FTIR spectrum of [Ni(SalPOHen)]; Figure S4: HRMS spectrum of [Ni(SalPOHen)]; Figure S5: ORTEP plot of [Ni(SalPOHen)]. Crystallographic data for [Ni(SalPOHen)] [50,51,52,53,54,55].
