# Supplementary material for: Tuning the Charge Transport in Nickel Salicylaldimine Polymers by the Ligand Structure
Source: Molecules. 2022 Dec 12;27(24):8798. doi: 10.3390/molecules27248798 (PMC9787065; doi:10.3390/molecules27248798)
Supplement: Supplementary file 1 [file molecules-27-08798-s001.zip › molecules-2046968-supplementary.pdf]

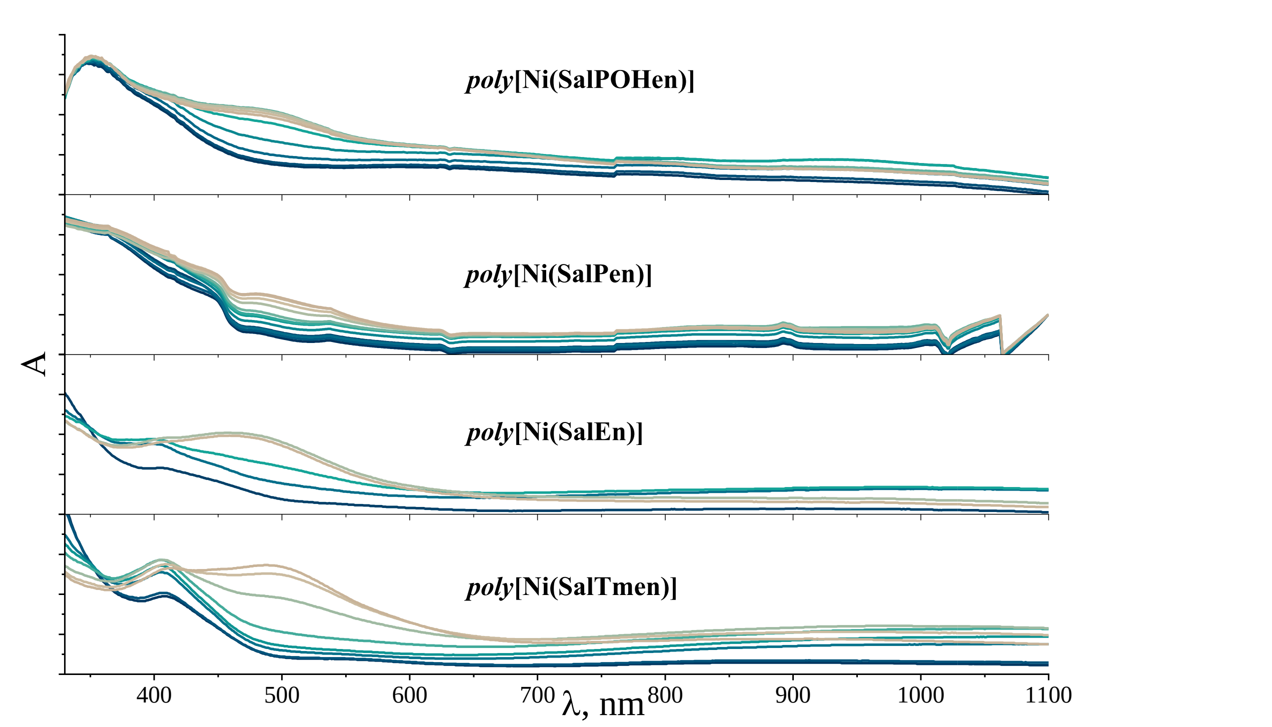


Figure S1. *Operando* UV-Vis spectra of the studied polymers.

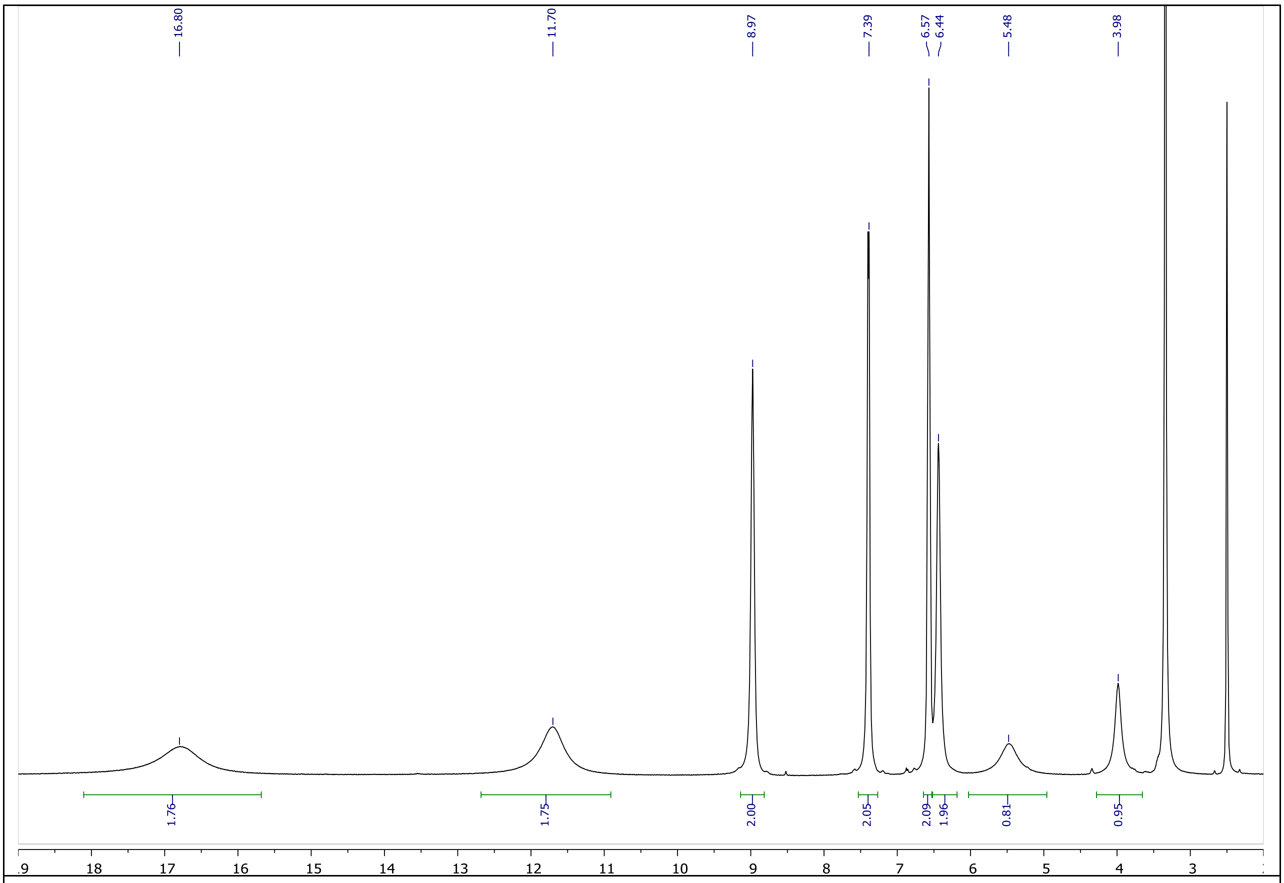


Figure S2. ¹H NMR spectrum of **[Ni(SalPOHen)]**

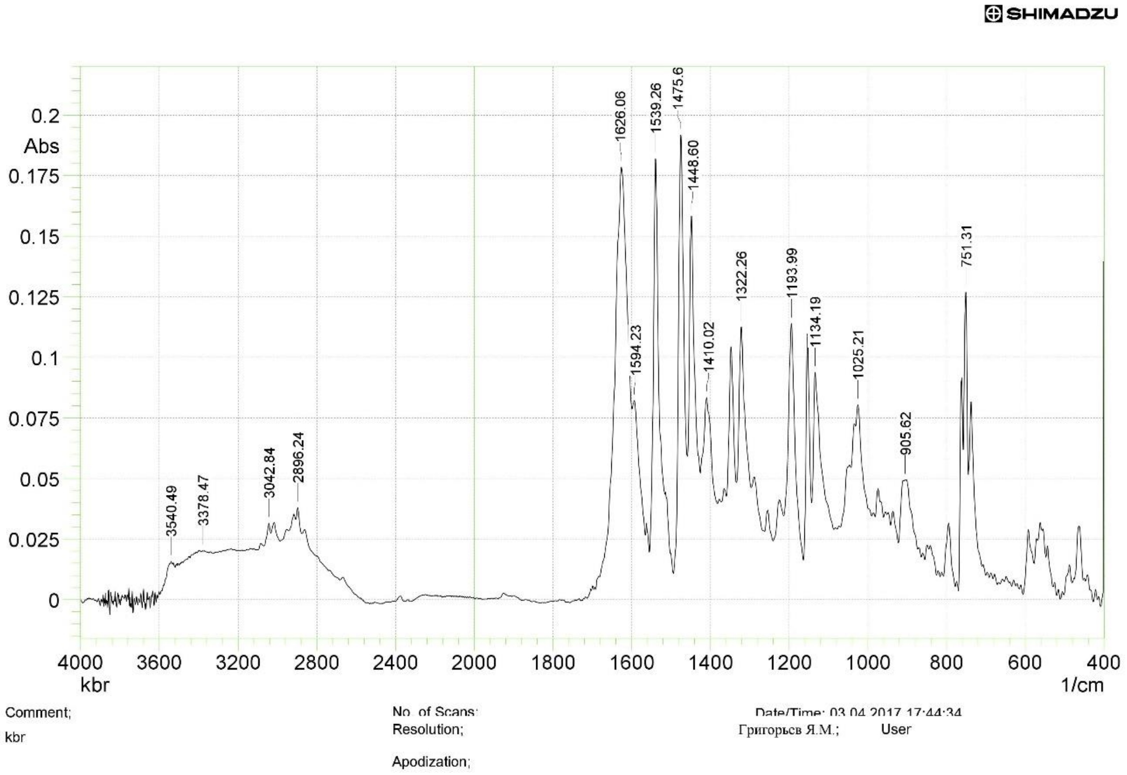


Figure S3. FTIR spectrum of **[Ni(SalPOHen)]**

# Display Report

**Analysis Info**

| | | | |
|---|---|---|---|
| Analysis Name | D:\Data\Work\2017\April\11\DAL_034_000001.d | Acquisition Date | 4/11/2017 11:09:38 AM |
| Method | tune_low.m | Operator | BDAL@DE |
| Sample Name | DAL_034_ | Instrument | maXis 62 |
| Comment | | | |

**Acquisition Parameter**

| | | | | | |
|---|---|---|---|---|---|
| Source Type | ESI | Ion Polarity | Positive | Set Nebulizer | 0.3 Bar |
| Focus | Not active | Set Capillary | 3200 V | Set Dry Heater | 180 °C |
| Scan Begin | 50 m/z | Set End Plate Offset | -500 V | Set Dry Gas | 4.0 l/min |
| Scan End | 3000 m/z | Set Collision Cell RF | 300.0 Vpp | Set Divert Valve | Source |

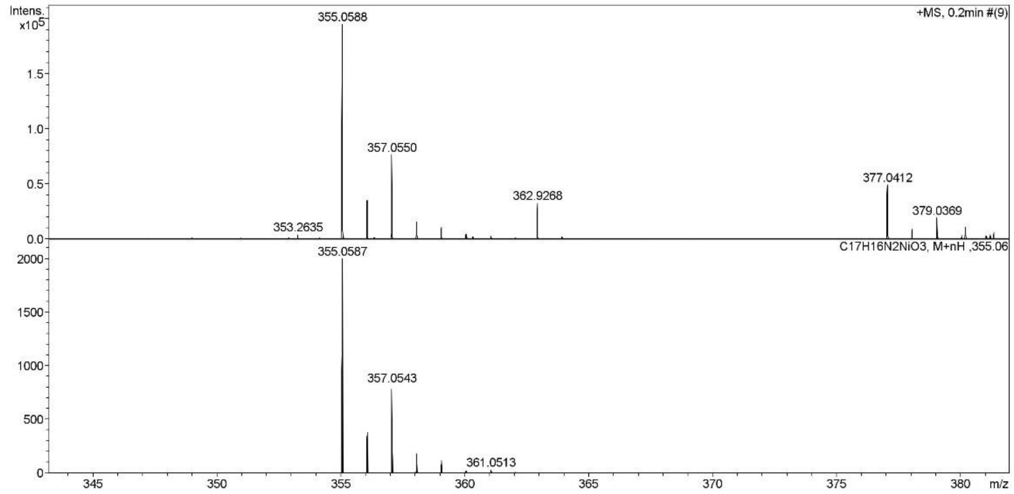


Bruker Compass DataAnalysis 4.0 printed: 4/12/2017 12:47:44 PM Page 1 of 1

Create PDF files without this message by purchasing novaPDF printer (http://www.novapdf.com)

Figure S4. HRMS spectrum of **[Ni(SalPOHen)]**

**Crystallographic data for [Ni(SalPOHen)]**

X-ray single crystal analysis were performed on Agilent Technologies «Supernova» diffractometer with monochromated Mo K$\alpha$ radiation. Crystal was measured at the temperature of 100 K. The structure has been solved by the Superflip[50–52] and ShelXS[53] structure solution programs using Charge Flipping and Direct Methods, respectively, and refined with the ShelXL[54] refinement incorporated in the OLEX$^2$ program package[55]. CCDC 1875699 contains the supplementary crystallographic data for this paper. These data can be obtained free of charge from The Cambridge Crystallographic Data Centre via http://www.ccdc.cam.ac.uk.

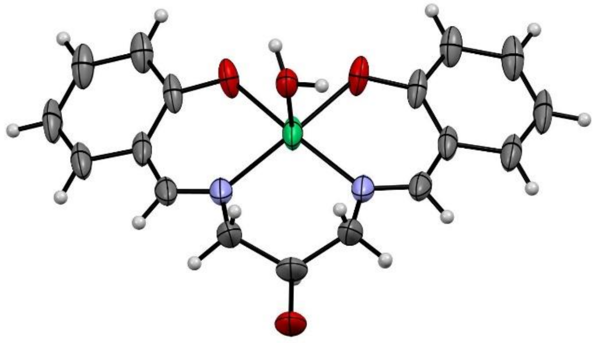

**Crystal Data** for $C_{34}H_{30}N_4Ni_2O_3$ ($M$ =740.04 g/mol): orthorhombic, space group Pnma (no. 62), $a$ = 8.6387(8) Å, $b$ = 23.9590(19) Å, $c$ = 7.5179(6) Å, $V$ = 1556.0(2) Å$^3$, $Z$ = 2, $T$ = 100(1) K, μ(MoK$\alpha$) = 1.271 mm$^{-1}$, $Dcalc$ = 1.579 g/cm$_3$, 6890 reflections measured (7.38° ≤ 2Θ ≤ 47.5°), 1824 unique ($R_{int}$ = 0.0741, $R_{sigma}$ = 0.1142) which were used in all calculations. The final $R_1$ was 0.0695 (I > 2σ(I)) and $wR_2$ was 0.1692 (all data).
CCDC 1503103

Figure S5. ORTEP plot of **[Ni(SalPOHen)]**
